# Multi-Scale Structural Insights into Enzymatically Hydrolyzed Lentil Starch Concentrates Prepared by In Vitro Method Using Different Types of Enzymes

**DOI:** 10.3390/foods12112150

**Published:** 2023-05-26

**Authors:** Namei Ren, Xinzhong Hu, Zhen Ma

**Affiliations:** College of Food Engineering and Nutritional Science, Shaanxi Normal University, Xi’an 710062, China; jenniena@163.com (N.R.); hxinzhong@snnu.edu.cn (X.H.)

**Keywords:** lentil, amylolysis, enzymatically hydrolyzed starch concentrates, molecular weight distribution, crystallinity, short- and long-range structure

## Abstract

This study was undertaken to investigate the enzymatic hydrolysis of lentil starch concentrates from conventional cooked seeds (CCLSC) by the action of different types of enzymes, including pancreatin (PC-EHSC), heat-stable α-amylase (HS-EHSC), β-amylase (βA-EHSC), amyloglucosidase (AMG-EHSC), and multi-enzymes (βA-HS-AMG-EHSC); their multi-scale structural characteristics of the enzymatic hydrolysis products of lentil starch concentrates were compared. The morphological features distinguished among different samples. The Fourier-transform infrared spectroscopy and solid-state ^13^C CP/MAS NMR spectral features indicated the possible formation of a binary and ternary complex among amylose, protein and lipids. The X-ray diffraction results revealed that the V-type characteristic diffraction peaks were more obvious for samples including PC-EHSC and βA-EHSC, which was in line with their lowest polydispersity index (DP_n_). PC-EHSC and βA-EHSC also showed an increased peak intensity of the scattering maximum on the small-angle X-ray scattering spectra, whereas CCLSC exhibited an overall lower peak intensity within the studied *q* range of scattering. The highest XRD crystallinity and the lowest DP_n_ value obtained for PC-EHSC indicated that the starch polymers modified by pancreatin could produce glucan chains with a comparatively homogenous *M*_w_ distribution that are readily recrystallized by hydrogen bonding through chain aggregation. Comparatively, the lowest relative crystallinity for HS-EHSC obtained from XRD suggested that thermostable α-amylolysis was unfavorable for the formation of starch structure with a higher degree of molecular order. This study could provide useful information for the needed research to obtain a deeper understanding of the impact of different amylolysis actions on the structural organization of starch hydrolysates and to provide a theoretical foundation for the development of fermentable enzymatically hydrolyzed starch with well-tailored physiological properties.

## 1. Introduction

Lentil, one of the important crops belonging to leguminaseae family, is botanically classified as *Lens culinaris*. Lentil seeds contain approximately 67–69% carbohydrates (44% of which are contributed by the presence of starch) and 24–30% protein [1]. The high ratio of amylose/amylopectin of lentil starch is associated with its low degree of crystallinity and gelatinization enthalpy [2]. Thermal processing of lentils has been extensively used to improve their nutritional, functional and sensory properties. Resistant starch (RS) is defined as the fraction of starch that is not accessible to the digestive enzymes in the small intestine of healthy individuals and reaches the colon for partial or complete fermentation. The resistant starch content in lentil seeds varied between 11.4% and 14.9% for different cultivars [3].

**Figure 1:**
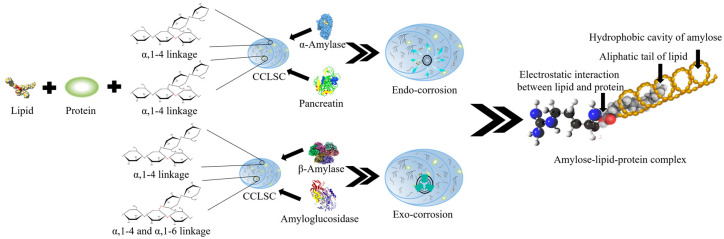
The schematic diagram of the amylolysis process of different enzymes on lentil starch concentrates obtained from the precooked seeds.

A number of processing methods including conventional cooking, extrusion, autoclaving, microwave cooking and acid hydrolysis have been applied to enhance the RS content of starch from different botanical origins [4]. Following the different processing treatments, different enzymes including porcine pancreatin, α-amylase, β-amylase, amyloglucosidase and a combination thereof have been used to collect enzymatically hydrolyzed starch concentrates (EHSC) [5]. Enzymatic hydrolysis of native and processed starch concentrates plays a significant role in various biological and industrial processing, making it an essential step in the applications of starch concentrates in both food and non-food industries [6]. Previous studies have revealed that in addition to the application of different processing conditions as mentioned above, the enzymatic hydrolysis process itself could also facilitate the rearrangement of amylose chains into an enzymatic-resistant structure with high crystallinity [7]. α-Amylase (α-1,4 glucan-4-glucanohydrolase, *E*.*C*. 3.2.1.1) randomly hydrolyzes the α(1→4) glycosidic linkages of starch concentrates substrate by an endo-action, with dextrin products having α-configuration at the anomeric carbon of the newly produced reducing end (Figure 1). α-Amylase that is from different natural sources such as mammals, microorganisms and plants, has different specificities with respect to the degradation products [8]. Thermo-stable α-amylase from bacteria mainly produces maltose and oligosaccharides with high molecular weight [5]. Porcine pancreatin contains a mixture of enzymes, principally pancreatic α-amylase, lipase and protease. The degradation products of pancreatic α-amylase are mainly composed of branched α-limit dextrins. Amyloglucosidase (AMG) is capable of hydrolyzing both α(1→4) and α(1→6) glycosidic linkages and converting oligomers produced from α-amylase digestion to glucose. However, the digestion of the highly branched starch substrate by AMG is considered as a slow rate-limiting step, since α-amylase itself has no debranching activity [9]. β-Amylase (*E.C.* 3.2.1.2) is an exo-hydrolase that specifically hydrolyzes the α(1→4) linkage of the two D-anhydroglucose monomers inward from the non-reducing end of starch polymers. The catalysis is able to produce a maltose each time hydrolysis is carried out, and is then stopped when an α(1→6) linkage or substitution group is encountered [10].

Factors including the molecular composition and structural characteristics of the starch substrate, the diffusion of the enzyme onto the substrate surface, the mechanism of enzyme action and its catalytic efficiency could all influence the kinetics of enzymatically hydrolyzed starch formation and consequently the fine molecular structure of starch hydrolysis products. Compared with native starch, the accessibility of the gelatinized starch substrate to enzymes is greater and not restricted by α-glucan associations such as double helices (especially arranged in crystallites) [11]. From the morphological point of view, the cracks and scale-like structure that appeared for the gelatinized starch, which was formed due to the swelling and retrogradation of starch granules, could also lead to higher accessibilities of enzymatic attack. Simultaneously, the composition of food matrices surrounding the starch granule and the role of the starch molecular interactions with proteins and lipids could both impact the diffusion of digestive enzymes onto the starch substrate [12]. Pre-cooking could expand the starch granules and separate amylose from the starch structure. Meanwhile, proteins are denatured during pre-cooking, and the hydrophobic groups (e.g., methionine and cysteine), which are normally concealed in the protein interior core, are then exposed. This could facilitate the non-polar interaction between amylose and protein in the lentil seeds. In addition, the endogenous lipids and proteins could also conjugate with starch and form binary and/or ternary complexes during cooking and the enzymatic hydrolysis process. The existence of both the starch granule-associated protein and the endogenous lipids that are attached to starch are, therefore, believed to affect the susceptibility of lentil starch concentrates to degradation with different types of enzymes and the structural features of starch hydrolysis products [13]. The structural characteristics of the hydrolysate products obtained after enzymatic hydrolysis in turn appear to have considerable implications on the host physiological response [4]. Although some information is available on the enzymatic digestion of starch from different botanical origins, the structural features of the hydrolysis products from starch concentrates using different types of enzymes and their comparison has not been fully understood. This study, therefore, was undertaken to investigate the enzymatic hydrolysis of conventionally cooked lentil starch concentrates by the action of different types of enzymes including pancreatin, thermostable α-amylase, β-amylase, amyloglucosidase (AMG), and multi-enzymes (heat-stable α-amylase, β-amylase and AMG). The multi-scale structural characteristics of the enzymatic hydrolysis products of the lentil starch concentrates was compared, with a view to providing useful information for needed research, in order to obtain a deeper understanding of the impact of different amylolysis action on the structural molecular rearrangement under the influence of protein and lipids and the potential interactions herein, as well as to provide a theoretical foundation for the development of fermentable enzymatically hydrolyzed starch concentrates with well-tailored physiological properties.

## 2. Materials and Method

### 2.1. Materials

The lentil seeds were from Daohuaxiang Co.^TM^ (Zaozhuang, Shandong Province, China). Thermostable α-amylase from *Bacillus licheniformis* (A3306, 5000 U/mL), β-amylase (A7130, 400 U/g), amyloglucosidase (10115, 70 U/mg) and pancreatin from porcine pancreas (P7575, 8 × USP specifications) were purchased from Sigma-Aldrich Chemical Co. (St. Louis, MO, USA). All other reagents used in this study were of analytical grade. 

### 2.2. Pre-Cooking of Lentil Seeds and Enrichment of Starch

The whole lentil seeds were conventionally cooked in boiling water (1:6, *w*/*v*) for 20 min, followed by freeze-drying and enrichment of lentil starch concentrates according to the method described by Miao et al. (2015) [14]. The obtained sample was referred to as lentil starch concentrates from conventional cooked seeds (CCLSC). 

#### Protein and Lipid Content of CCLSC

The crude protein content of CCLSC was measured by the nitrogen combustion method using a nitrogen analyzer (LECO Corporation, St Joseph, MN, USA). A nitrogen conversion factor of 5.40 was used to calculate the crude protein content. The total lipid content of CCLSC was Soxhlet extracted in a Buchi B-811 extractor (Wanjie Technology Co., Ltd., Shanghai, China) with petroleum ether, at 80 °C. The amount of total lipids was calculated as a sum of the extracted lipids after the evaporation of organic solvent from the recipients and drying to a constant weight. The protein and lipid contents of CCLSC were 17.96% ± 0.7281 and 1.08% ± 0.0954, respectively.

### 2.3. Enzymatic Modification 

#### 2.3.1. Enzymatic Hydrolysis by β-Amylase

Ten grams of CCLSC was dispersed in 100 mL sodium acetate buffer (100 mM, pH 5.0), and β-amylase (0.5%, d.w. of starch) was then added to the dispersion. Enzymatic hydrolysis was performed in a thermostatic oscillator (SHA-B, Chenghui Instrument, Jiangsu, China) at 55 °C for 12 h (128 rpm). Immediately after the hydrolysis, the same volume of 95% ethanol was added to terminate the reaction. The collected sample was centrifuged at 3000× *g* for 10 min, and the sediment was washed with distilled water for three times before oven drying at 42 °C, grounding and sieving (149 μm). The obtained starch sample was referred to as βA-EHSC (enzymatically hydrolyzed starch concentrates obtained by the treatment with β-amylase). 

#### 2.3.2. Enzymatic Hydrolysis by Thermostable α-Amylase

Ten grams of CCLSC was dispersed in 20 mL sodium acetate buffer (100 mM, pH 6.0) containing 0.5 mL thermostable α-amylase, and the enzymatic hydrolysis was performed in a thermostatic oscillator at 95 °C for 1 h (128 rpm). The obtained sample was then processed following the same procedure as described above (Section 2.3.1), and the obtained starch sample was defined as HS-EHSC (enzymatically hydrolyzed starch concentrates obtained by the treatment with heat-stable α-amylase).

#### 2.3.3. Enzymatic Hydrolysis by Pancreatin

A sample of CCLSC (1.5 g) was slurried in 30 mL sodium acetate buffer (100 mM, pH 5.0) and was then hydrolyzed by pancreatin at 37 °C for 16 h. The obtained sample was processed following the same procedure as described above (Section 2.3.1) and the obtained starch sample was defined as PC-EHSC (enzymatically hydrolyzed starch concentrates obtained by the treatment with pancreatin).

#### 2.3.4. Enzymatic Modification by Amyloglucosidase

A sample of CCLSC (1.5 g) was dispersed in sodium acetate buffer (100 mM, pH 4.5), followed by the addition of amyloglucosidase. The enzymatic hydrolysis was performed at 60 °C for 72 h. The obtained sample was processed following the same procedure as described above (Section 2.3.1), and the obtained starch sample was defined as AMG-EHSC (enzymatically hydrolyzed starch concentrates obtained by the treatment with amyloglucosidase).

#### 2.3.5. Enzymatic Hydrolysis by the Action of Multi-Enzymes

A sample of CCLSC (1.5 g) was suspended in sodium acetate buffer (100 mM, pH 5.0) and then hydrolyzed by β-amylase for 12 h at a constant temperature of 55 °C. The pH of the suspension was adjusted to 6.0, followed by the addition of thermostable α-amylase. The entire hydrolysis process was continued for 1 h at 95 °C in a thermostatic oscillator (128 rpm). The pH of the obtained solution was then adjusted to 4.5, followed by the addition of amyloglucosidase, and the hydrolysis was then conducted for 72 h at 60 °C. The obtained sample was processed following the same procedure as described above (Section 2.3.1) and the obtained starch sample was defined as βA-HS-AMG-EHSC (enzymatically hydrolyzed starch concentrates obtained by the treatment with β-amylase, heat-stable α-amylase and amyloglucosidase).

### 2.4. Scanning Electron Microscopy (SEM)

The morphological features of CCLSC and EHSC samples prepared by different enzymatic treatments were obtained using a Quanta 200 instrument (FEI company, Portland, OR, USA) at an accelerating voltage of 20 keV. The powder samples were imaged after fixing on a conductive adhesive and sputter coating with gold using an ion sputtering instrument (BAL-TEC, SCD005, Beijing Oubotong Optics Technology Co., Ltd., Beijing, China). The magnification was set at 1500×.

### 2.5. High-Performance Size-Exclusion Chromatography (HPSEC) 

The dry powder of CCLSC and different EHSC samples (2 mg) was dissolved in 1 mL dimethylsulfoxide containing 0.5 wt% LiBr and was incubated at 80 °C for 12 h. A size-exclusion chromatography instrument (Agilent 1260 series, Agilent Technologies, Waldbronn, Germany) equipped with a refractive index detector (RI, Optilab T-rEX, WYATT Corp., Santa Barbara, CA, USA) and a multi-angle laser light scattering detector (MALLS, DAMN HELEOS-II, WYATT Corp., Santa Barbara, CA, USA) was used to measure the molecular weight distribution of the studied samples, following the method of Yin et al. (2018) [15].

### 2.6. X-ray Diffraction (XRD)

The crystalline properties of CCLSC and EHSC samples were analyzed by an X-ray diffraction system (D/Max2550VB+/PC, Rigaku Corporation, Rigaku, Japan), following the method of Yin et al. (2018) [15]. The X-ray diffractogram was obtained at a scanning speed of 5°/min and a step size of 0.02. The resulting XRD spectra were analyzed using JADE 5.0 software (Materials Data Inc., Livermore, CA, USA) to calculate the percentage of relative crystallinity (C1).

### 2.7. Fourier-Transform Infrared (FTIR) Spectroscopy

Samples of CCLSC and different EHSC were blended with pre-dried KBr powder at a mass ratio of 1:200 and pressed into tablets before the measurement. The spectra were achieved using an FTIR spectrometer (TENSOR27; Bruker Optics GmBH, Ettlingen, Germany) at a resolution of 4 cm^−1^ from 400–4000 cm^−1^.

### 2.8. Solid-State Nuclear Magnetic Resonance (^13^C NMR)

The solid-state cross-polarization and magic-angle spinning nuclear magnetic resonance (^13^C CP/MAS NMR) measurement was carried out using an AVANCE III 400 MHz WB NMR spectrometer (Bruker Inc., Billerica, MA, USA) equipped with a double resonance H/X CP-MAS 4mm probe. The spectrometer was operated at a frequency of 100.62 MHz and the samples were spun at a rate of 6 kHz. The ^13^C NMR parameters, including crystallinity (C2), double helix content and the proportion of amorphous region (PPA) were quantified using PeakFit software (version 4.12, SeaSolve Inc., Framingham, MA, USA) following the method of Ren et al. (2021) [2].

### 2.9. Small Angle X-ray Scattering (SAXS)

The SAXS measurement was carried out on a SAXS system (Nanostar, Bruker AXS GmbH, MA, USA) at the sample concentration of 50%, *w*/*w*), following the method of Yin et al. (2018) [15]. The X-ray was generated from Cu–Kα radiation at 50 kV and 600 μA, with the wavelength of 1.54 Å. The power law exponent (α) was obtained according to the power law equation at the low *q* region of the SAXS spectra (Equation (1)). The self-similarity of the scattering object was characterized by the concept of the fractal; the relationship between the fractal dimension (surface/mass) and α values is illustrated by Equations (2) and (3). The SAXS data was also analyzed using the 1D correlation function (Equation (4)), and the average thickness of the crystalline layer (*d*_c_) was thereby calculated.

(1) $$I\left(q\right)\sim q^{-\alpha }$$

(2) $$D_{m}=-\alpha \;(-3<\alpha <-1)$$

(3) $$D_{s}=6+\alpha \;(-4<\alpha <-3)$$

(4) $$r=\frac{\int_{0}^{\infty }I\left(q\right)q^{2}\cos\left(qz\right)dq}{\int_{0}^{\infty }I\left(q\right)q^{2}dq}$$

where *I* is the intensity of scattering; *q* is the magnitude of the scattering vector defined as *q* = (4πsin*θ*)/*λ* (*λ* is the wavelength of the incident radiation, 2*θ* is the scattering angle and is half the angle through which the radiation is scattered); *α* is the power law exponent which reflects the mass/surface fractal dimensions of starch; *D_m_* and *D_s_* refer to mass fractal dimension and surface fractal dimension, respectively; *r* is the distance in real space; *d* is the long period (lamellar repeat distance) which can be determined as the *z* at the second maximum of *r*(*z*). 

### 2.10. Statistical Analysis

All data were statistically analyzed by analysis of variance (ANOVA) followed by Duncan’s test at *p* ≤ 0.05 using DPS software (Version 7.05, Wiley-Blackwell, Hoboken, NJ, USA).

## 3. Results and Discussion

### 3.1. Morphological Features Obtained by Scanning Electron Microscope 

**Figure 2:**
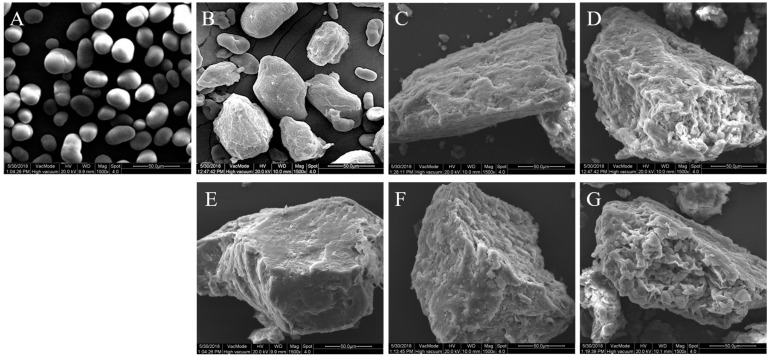
Scanning electron microscope images of native lentil starch concentrates (LSC), lentil starch concentrates from conventional cooked seeds (CCLSC) and enzymatically hydrolyzed starch concentrate residues obtained by different enzymatic hydrolysis methods (EHSC). (**A**) LSC: native lentil starch concentrates; (**B**) CCLSC: lentil starch concentrates from conventional cooked seeds; (**C**) PC-EHSC: enzymatically hydrolyzed starch concentrates obtained by the treatment with pancreatin; (**D**) AMG-EHSC: enzymatically hydrolyzed starch concentrates obtained by the treatment with amyloglucosidase; (**E**) βA-HS-AMG-EHSC: enzymatically hydrolyzed starch concentrates obtained by the treatment with β-amylase, heat-stable α-amylase and amyloglucosidase; (**F**) βA-EHSC: enzymatically hydrolyzed starch concentrates obtained by the treatment with β-amylase; (**G**) HS-EHSC: enzymatically hydrolyzed starch concentrates obtained by the treatment with heat-stable α-amylase.

The scanning electron microscope images of CCLSC and enzymatically hydrolyzed residues obtained by different enzymatic hydrolysis methods of lentil starch concentrates from precooked seeds are presented in Figure 2. Pre-cooking of lentil seeds resulted in a pronounced deformation of the granular morphology, with the appearance of enlarged chunks of aggregates with differing sizes and shapes. All the EHSC samples still retained the majority of the morphological features of gelatinized starch granules, due to the conventional cooking pretreatment of the lentil starch concentrates, showing the appearance of chunks of starchy mass embedded in the irregular shapes. Specifically, PC-EHSC and βA-HS-AMG-EHSC were more homogenous and smoother, with some fine holes occurring on the surface. Comparatively, the residues of the digested AMG-EHSC and βA-EHSC appeared to be different, and holes on the surface of the hydrolyzed particles could be seen in these samples. The hydrolysis of the lentil starch concentrates obtained from the precooked seeds with heat-stable α-amylase was distinguished by the formation of deeper holes along the grooves on the granular surface, which are connected to the interior channels. It was assumed that the high temperature applied during enzymatic hydrolysis using thermostable α-amylase could facilitate starch gelatinization and enable a higher susceptibility of the less organized starch chains toward α-amylase attack, leading to a more extensive corrosion by the enzymes. 

### 3.2. Molecular Weight by HPSEC

High-performance size-exclusion chromatography (HPSEC) analysis was performed to quantify the molecular weight distribution of EHSC obtained by different enzymatic hydrolysis methods, and the results are shown in Table 1. The trend of *M*_w_ values was in the following order: CCLSC > AMG-EHSC > βA-EHSC > βA-HS-AMG-EHSC > PC-EHSC > HS-EHSC, whereas the values of DP_n_ (polydispersity index) showed a slight discrepancy, viz., CCLSC > HS-EHSC > AMG-EHSC > βA-HS-AMG-EHSC > βA-EHSC > PC-EHSC. In comparison to CCLSC, the markedly decreased *M*_w_ and DP_n_ values upon amylolysis signified that the different enzymatic treatments could produce the glucan molecules with much shorter and more homogeneous molecular chains. Although many factors could influence the rate of enzymatic hydrolysis of starch concentrates, the accessibility of enzymes to the glycosidic bonds in the substrate was a major determinant [16]. Amyloglucosidase (AMG) is an exo-amylase that cleaves both the α(1→4) and α(1→6) linkages of amylose and amylopectin chains of the starch substrate (Figure 1) [17]. However, evidence has shown that AMG was more effective in hydrolyzing the α(1→4) linkage, which was 500 times faster than breaking the α(1→6) glycosidic bonds [9]. It was thus inferred that the number of α(1→6) branch linkages in the cluster physically hindered the enzyme diffusion and could be the rate-limiting factor for hydrolyzing the highly branched starch substrate, correlating with a low degree of enzymatic hydrolysis and consequently a relatively high *M*_w_ value obtained for AMG-EHSC, compared to other EHSC samples.

β-Amylase is an exo-amylase that is responsible for hydrolyzing the α(1→4) linkages of two D-anhydroglucose monomers inwards from the non-reducing ends of the glucan chain [18]. According to previous studies [18], the associated α(1→6) branch linkages in the cluster of starch substrate could act as a barrier against the binding to active sites of β-amylase, and the hydrolysis was terminated when the amount of glucose was between two and three units from the branching point, which partially trimmed the outer branches of starch molecules into new limiting dextrin. This could explain the relatively high *M*_w_ value obtained for βA-EHSC (5.690 × 10^4^ g/mol) compared to other samples, including PC-EHSC with a *M*_w_ value of 3.309 × 10^4^ g/mol and HS-EHSC with a *M*_w_ value of 1.711 × 10^3^ g/mol. 

A relatively low *M*_w_ value of 3.309 × 10^4^ was noticed for PC-EHSC. A possible reason was based on the postulation that, apart from the existing amylose-lipid complex in lentil starch concentrate granules, the endogenous lipids and free proteins would also conjugate with the amylose/amylopectin chains which became more mobile and available for complexation during pre-gelatinization [19]. After 16 h of incubation with pancreatin, the protease and lipase of pancreatin was able to assist in the enzymatic hydrolysis of conjugated proteins and lipids, which somewhat reduced their partial shielding effect and hence resulted in a deep hydrolysis of the polymer substrate [8]. The lowest DP_n_ value for PC-EHSC (1.037), which was closer to 1, indicated the higher homogeneity and narrower distribution of molecular weight in the starch concentrate samples treated with pancreatin.

For HS-EHSC, the high incubation temperature applied caused the disruption of the retrograded lentil starch concentrate, leading to an enhanced accessibility of heat stable α-amylase to the polymer substrate and a lower value of *M*_w_ (1.711 × 10^3^ g/mol) for HS-EHSC. Meanwhile, the enzymatic hydrolysis by heat stable α-amylase resulted in a wider molecular distribution of the polymer substrate, corresponding to its higher obtained DP_n_ value of 1.262.

### 3.3. Crystalline Structure

#### 3.3.1. XRD Analysis

**Figure 3:**
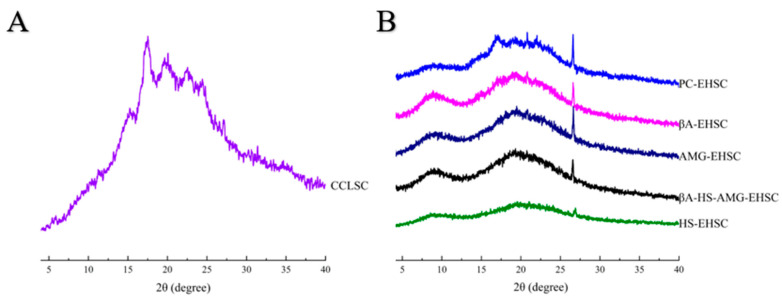
X-ray diffraction pattern of CCLSC and enzymatically hydrolyzed starch concentrate residues obtained by different enzymatic hydrolysis methods. (**A**) X-ray diffraction pattern of CCLSC; (**B**) X-ray diffraction pattern of enzymatically hydrolyzed starch concentrates. CCLSC: lentil starch concentrates from conventional cooked seeds; PC-EHSC: enzymatically hydrolyzed starch concentrates obtained by the treatment with pancreatin; βA-EHSC: enzymatically hydrolyzed starch concentrates obtained by the treatment with β-amylase; AMG-EHSC: enzymatically hydrolyzed starch concentrates obtained by the treatment with amyloglucosidase; βA-HS-AMG-EHSC: enzymatically hydrolyzed starch concentrates obtained by the treatment with β-amylase, heat-stable α-amylase and amyloglucosidase; HS-EHSC: enzymatically hydrolyzed starch concentrates obtained by the treatment with heat-stable α-amylase.

Figure 3 illustrates the XRD patterns of CCLSC and different EHSC residues obtained by different enzymatic hydrolysis methods of cooked lentil starch concentrates. It can be clearly seen that all EHSC samples showed a wide peak at 2*θ* = 8.9° and a sharp peak at 26.6°, both of which were characteristic diffraction peaks of the A-type crystalline structure. An absorption peak at 22.0°, which resembled a typical B-type, was also observed for all studied samples. The additional peaks at 19.0° and 20.8°, derived from the V-amylose helical structure in the presence or the absence of a lipid ligand, and a typical A-type diffraction peak centered at 17.1°, derived from starch recrystallization, were also presented on the X-ray diffractograms for PC-EHSC, βA-EHSC and AMG-EHSC. The observation validated the fact that all EHSC samples consisted of a C_A_ + V crystalline polymorph. The V-amylose structure is a single, left-handed helix with an internal cavity where the complexed ligand can reside. In the lamellar structural architecture of different EHSC residues, the crystalline regions consisting of A-, B- and V-type crystal polymorphs make up the lamellar region, whereas the amorphous un-complexed amylose components associated with amylose fractions with small molecular size under the action of enzymatic hydrolysis are arranged in another region that is unable to diffract X-rays [20]. In addition, some other peaks at 2*θ* = 7.5° and 21.3° that are responsible for the un-complexed fatty acids were also noticed on the X-ray diffraction pattern. The diffractogram of CCLSC was characterized by the reflections at the spectral diffraction angles in the position of 15.1°, 17.1°, 19.0°, 22.0°, 23.7° and 26.6°, which confirmed that CCLSC contains domains of the type C and type V crystalline structure. A proportion of the B-type crystalline peaks of the pre-cooked lentil starch concentrates vanished in the EHSC samples, possibly due to the preferential hydrolysis of branch points of B-type starch concentrates, which were clustered in the amorphous domains after CCLSC being subjected to several enzymatic treatments [21]. Based on the study of Dhital, et al. (2014) [22], the regions with a less organized molecular structure of starch granules happen to be the potential catalytic binding sites for α-amylase. The glucosyl hydroxyl groups of the α-1,4 linked glucan chains are distributed on the outside surface of the amylose helix, leaving the inner core as a more hydrophobic cavity lined with methylene groups and glycosidic oxygens, which can form inclusion complexes with free fatty acid chains [23] and are responsible for the distinctive V-type patterns on the X-ray diffractogram. The formation of the V-type crystalline structure in this study could be both derived from the pre-cooking of the LSC and the enzymatic hydrolysis process. During pre-cooking, the proteins developed a continuous network by cross-linking, and amylose and amylopectin were expanded and separated from the swollen granules and further dispersed within the protein network, followed by the leaching of lipid into the continuous phase, which accelerated the complexation of amylose with lipids and proteins by hydrophobic and electrostatic interaction, and consequently lead to an increased structural order [24]. Meanwhile, during the enzymatic hydrolysis process, more linear amylose molecular chains with favorable DP_n_ could be generated, which increased the availability of the released linear *α*(1–4)-glucan chains incorporating into the V-type inclusion complex [25]. Studies have shown that there is an optimal linear glucan chain length that facilitates the formation of complexes with lipids. Enzymes that hydrolyze amylose or debranch amylopectin can increase the amounts of linear glucans with a chain length in the optimum range. The conformation of amylose will change into a single helix with a hydrophobic cavity when it is gelatinized in the presence of lipids, and the hydrophobic cavity formed by the depolymerized glucan chains can then interact with the hydrophobic ligand and form complexes, such as the starch-lipid complex (V-complexes). It was worth noting in Figure 3 that the V-type characteristic diffraction peaks were more obvious for samples including PC-EHSC and βA-EHSC. This observation is in line with the lowest DP_n_ values obtained for PC-EHSC (DP_n_ = 1.037) and βA-EHSC (DP_n_ = 1.038), suggesting that their starch chains with a relatively homogenous molecular weight distribution might be more effective in participating in the formation of the V-type inclusion complex with free fatty acids in pre-cooked lentil starch concentrates. Liu et al. (2019) [25] also demonstrated that when β-amylase treatment was applied to potato and maize starches, the intensity of the V-type XRD crystalline pattern was increased.

It was also especially noticed that HS-EHSC displayed a relatively flat and broad reflection in the 2θ range, between 15° and 26°. The excessive hydrolysis of pre-cooked lentil starch concentrates during enzymatic treatment with thermostable α-amylase could generate shortened polymer chains including oligosaccharides with varying lengths and branched oligosaccharides (viz., limit dextrins), which are not favorable for folding and organizing into an orderly, packed crystalline structure. This assumption was also supported by the relatively low *M*_w_ value obtained for HS-EHSC during the HPSEC analyses. Based on the studies of Gelders et al. (2004) [26], if the amylose chains are too short, they cannot be arranged into ordered V-type amylose complexes. According to Mutungi et al. (2012) [27], these non-crystalline interfaces could induce reflection fluctuations in the relative spatial arrangement between corresponding atoms in the ideal lattices, leading to the decreased scattering ability of crystallites. In addition, small crystallites that have only a few lattice planes might not produce adequately detectable diffraction intensities. 

From Table 1, it can be seen that CCLSC and various EHSC samples exhibit different relative crystallinity levels (C1), which are in the following order: PC-EHSC > AMG-EHSC > βA-HS-AMG-EHSC > CCLSC > βA-EHSC > HS-EHSC. The highest C1 value was noticed for PC-EHSC. This could be explained by the assumption that the removal of protein and lipids in cooked starch concentrates treated with pancreatin promoted the crystallinity of the samples. This matched the relatively narrower distribution of molecular weight (3.309 × 10^4^ g/mol) of PC-EHSC, indicating that the starch polymers modified by pancreatin could produce glucan chains with a comparatively homogenous *M*_w_ distribution that are readily recrystallized by hydrogen bonding through chain aggregation [28]. On the other hand, the lowest C1 value for HS-EHSC suggested that thermostable α-amylolysis was unfavorable for the formation of starch structure with a higher degree of molecular order. This could be ascribed to the high temperature involved during the preparation of HS-EHSC, which most likely to result in an extensive enzymolysis of the starch concentrate granular structure. According to a previous study [10], the higher degree of enzymatic degradation could lead to the production of a higher proportion of short-branched chains and a lower proportion of long-branched chains. Simultaneously, the production of short-branched chains made recrystallization more difficult, which was in line with the relatively lower C1 value obtained for HS-EHSC by XRD. Considering the effect of β-amylase by trimming the amylose and the outer branches of the amylopectin molecules, the XRD results revealed that starch chains with such changes also correlated with a relatively low ability to reassociate into the long-range crystal structure, corresponding to the second lowest C1 value obtained (Table 1).

#### 3.3.2. FTIR Analysis 

**Figure 4:**
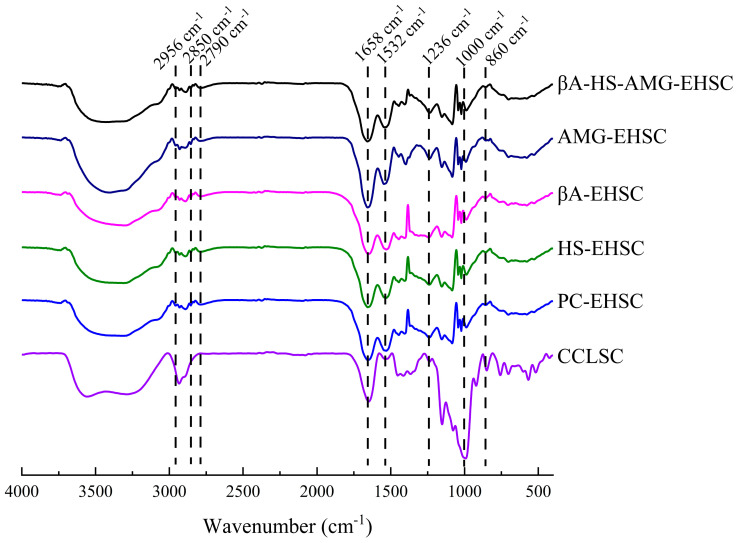
FTIR spectra of CCLSC and enzymatically hydrolyzed starch concentrate residues obtained by different enzymatic hydrolysis methods. CCLSC: lentil starch concentrates from conventionally cooked seeds; PC-EHSC: enzymatically hydrolyzed starch concentrates obtained by the treatment with pancreatin; HS-EHSC: enzymatically hydrolyzed starch concentrates obtained by the treatment with heat-stable α-amylase; βA-EHSC: enzymatically hydrolyzed starch concentrates obtained by the treatment with β-amylase; AMG-EHSC: enzymatically hydrolyzed starch concentrates obtained by the treatment with amyloglucosidase; βA-HS-AMG-EHSC: enzymatically hydrolyzed starch concentrates obtained by the treatment with β-amylase, heat-stable α-amylase and amyloglucosidase.

The FT-IR data were collected for the EHSC samples obtained by different enzymatic treatments in the present study from 4000–400 cm^−1^, and the spectra are shown in Figure 4. A wide and strong absorption peak in the range of 3000–3700 cm^−1^ was observed for each sample, corresponding to the inter- and intra-molecular stretching related to the O–H and N–H vibration of the EHSC samples [29]. The bands at 995, 1080 and 1160 cm^−1^ were susceptible to the C–O and C–C stretching vibrations, and the wavenumber of 860 cm^−1^ was ascribed to the C–O–C glycosidic band in the EHSC [30]. A decreased intensity of the absorption peak centered at ~1000 cm^−1^ for different EHSC samples in contrast to CCLSC suggested the efficient cleavage of glycosidic bonds during different enzymatic hydrolysis treatments. 

The protein secondary structure in the range of 1200–1700 cm^−1^ is also observed in Figure 4. In detail, the Amide *I* band at 1658 cm^−1^ was associated with the C=O stretching in the proteins secondary structure, the Amide *II* band at 1448 and 1532 cm^−1^ was linked to the C–N and N–H bending, whereas the absorption at 1236 cm^−1^ (Amide *III* band) was related to the C–H vibration in the peptide bonds. All the above three amide characteristic bands were observed in the starch concentrate samples in the present study, suggesting the existence of protein in the EHSC, with enzymatically hydrolyzed starch concentrates obtained by the treatment with heat-stable α-amylase samples being subjected to cooking and enzymolysis treatments. Individual peaks shown on the spectra in the Amide *I* region (1700–1600 cm^−1^) were classified as α-helix (1650–1658 cm^−1^), β-sheet (1620–1635 cm^−1^) and random structure (1640–1650 cm^−1^) [31]. Protein aggregation that was expected to occur during the pre-cooking of lentil seeds was also observed at 1615 and 1685 cm^−1^ on the FT-IR spectrum [32]. It was noticed that the intensity of the Amide *II* band at 1532 cm^−1^ which is linked to N–H bending and the peak at 2790 cm^−1^ which is associated with the asymmetric hydrogen bonding of carboxylic acid were markedly increased after different enzymatic treatments, compared with the CCLSC. This observation suggested that the electrostatic interaction could be formed between the negatively charged carboxyl group of lipids and positively charged amino group of proteins during enzymatic hydrolysis, which might act as an important force for the additional development of the ternary complex through hydrophobic interaction between amylose and the aliphatic tail of fatty acids [33].

It was notable that three characteristic peaks for lipids appeared at 2956 cm^−1^, 2850 cm^−1^ and 1745 cm^−1^, corresponding to the CH_3_ asymmetric stretch, CH_2_ symmetric stretching vibration, and C=O stretch vibration of fatty acids, respectively [29,34]. The hydrophobic interaction between the interior part of amylose helices and the aliphatic tail of free fatty acids, as well as the electrostatic interaction between the negatively charged carboxyl groups of fatty acids and polyionic protein have both been proved to be the important forces during the formation of the amylose-lipid complex [35]. In comparison to CCLSC, the intensification of the band at 2956 cm^−1^ and 2850 cm^−1^ with the appearance of more tiny and distinguished peaks for the enzymatically hydrolyzed samples could be observed (Figure 4). It was speculated that such a change in the carbonyl stretch vibration pertaining to fatty acids could be due to the changes in the conformational and physical state of lipids when complexed with starch and/or protein [36].

The EHSC samples had peaks at 2854 cm^−1^ and 1578 cm^−1^, indicating that protein and lipids had been complexed [37]. The appearance of the absorption bands at ~2852 cm^−1^ and ~2930 cm^−1^ on the FT-IR spectra inferred that the spatial structure of protein attached to starch, overlapping the methylene symmetric stretching vibration of the protein macromolecules, has been unfolded [38]. Furthermore, a blueshift of the sharp peak at 1532 cm^−1^ was noted in the case of AMG-EHSC and βA-HS-AMG-EHSC, suggesting that the N–H bonding tended to be more stable in these samples. To further study the short-range, ordered structure of the EHSC samples obtained by different enzymatic treatment methods, the intensity ratios at 1047/1022 cm^−1^ and 986/1022 cm^−1^ were calculated. The absorbance ratio at 1047/1022 cm^−1^ was sensitive to alternations in short-range crystallinity and degree of ordering (DO) of the EHSC samples, whereas the absorbance ratio at 986/1022 cm^−1^ reflected the degree of double helix (DD) [39]. As shown in Table 1, the trend in DD and DO values were both consistent with those observed for C1 values, which was in the following order: PC-EHSC > AMG-EHSC > βA-HS-AMG-EHSC > CCLSC > βA-EHSC > HS-EHSC.

### 3.4. Structural Order by Solid-State ^13^C CP MAS NMR on the Molecular Level

**Figure 5:**
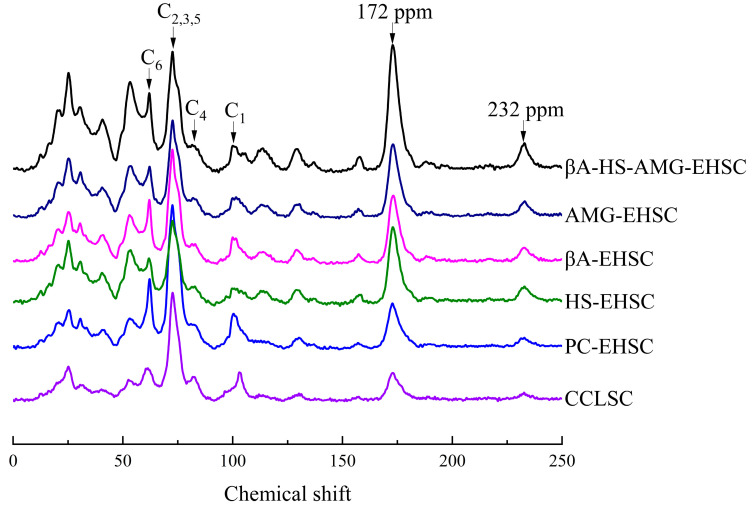
The ^13^C CP/MPS NMR spectra of CCLSC and enzymatically hydrolyzed starch concentrate residues obtained by different enzymatic hydrolysis methods. CCLSC: lentil starch concentrates from conventional cooked seeds; PC-EHSC: enzymatically hydrolyzed starch concentrates obtained by the treatment with pancreatin; HS-EHSC: enzymatically hydrolyzed starch concentrates obtained by the treatment with heat-stable α-amylase; βA-EHSC: enzymatically hydrolyzed starch concentrates obtained by the treatment with β-amylase; AMG-EHSC: enzymatically hydrolyzed starch concentrates obtained by the treatment with amyloglucosidase; βA-HS-AMG-EHSC: enzymatically hydrolyzed starch concentrates obtained by the treatment with β-amylase, heat-stable α-amylase and amyloglucosidase.

The solid-state ^13^C CP/MAS NMR spectra of the EHSC samples obtained by different enzymatic treatments are illustrated in Figure 5. ^13^C CP/MAS NMR was used to measure the structural order in the amylolyzed lentil starch concentrates on a molecular level, in contrast to the XRD analysis on a crystalline level. The resonance parameters obtained from the solid-state NMR spectra could also reflect the behavior of each component, including protein, starch and lipids in the multi-component system [40]. Spectral features included the aliphatic carbons with the contribution from lipids detectable at 10–40 ppm and 52 ppm and *O*-alkyl carbons starch at 60–110 ppm, aromatic carbons visible at 130 ppm, and carbonyl carbons of proteins at 172 ppm [34,41]. The major resonances of the starch component at 95–105 ppm, 80–84 ppm, and 58–65 ppm coincided with those of C1, C4, and C6 of hexapyranoses, respectively, whereas the overlapping signal centered at 68–78 ppm was assigned to C2, C3, and C5.

The quantification of the ^13^C NMR crystallinities, double helix content and proportion of amorphous phase (PPA) with respect to different enzymatic treatments, is presented in Table 1. It can be clearly seen that C2 followed the order of PC-EHSC > βA-HS-AMG-EHSC > βA-EHSC > CCLSC > AMG-EHSC > HS-EHSC. Upon the different amylolytic hydrolysis methods applied in this study, a pronounced downfield ^13^C chemical shift of the signal for C1 of the hexapyranose unit of CCLSC was especially noticed in Figure 5, suggesting the formation of a V-type amylose-lipid complex. This was supported by previous studies, with the conformational change being detected on the ^13^C NMR spectra, due to the formation of the amylose-lipid complex, particularly due to (i) the changes in the torsion angles of the glycosidic linkages, resulting in a different pattern of electron distribution, and (ii) the structural alteration of the amylose molecule from a random coil to a helix [42]. This observation was consistent with the XRD analysis (Figure 3). It can be clearly seen from Table 1 that the proportion of double helix has decreased to varying extents upon different enzymatic treatments. It was proposed that, during the amylolysis process, instead of being converted into amorphous mass, the double helices in CCLSC might be disassociated into single chains. Meanwhile, a high proportion of glucans with a reduced chain length could be generated, which further promote the aggregation of amylose with endogenous lipids and proteins.

Compared with CCLSC, the resonances at 10–40 ppm with the contribution from lipids and at 172 ppm with the contribution from proteins showed the greatest change amongst all signal peaks after different enzymatic treatments, whereas the peak intensities were markedly higher upon the enzymatic hydrolysis treatments. Furthermore, an up-field shift of the resonance at 172 ppm was also observed for the amylolytic hydrolyzed starch concentrates compared, with CCLSC. This observation suggested that the different enzymatic treatments applied could induce several consecutive changes involving the hydrolysis of glycosidic bonds, the disruption of the molecular order of CCLSC by the breaking of the hydrogen bonds, and the self-rearrangement of starch chains and starch-protein/lipids complex formation. Additionally, in comparison to CCLSC, the broadening of the resonance band at 172 ppm observed in all the EHSC samples might be attributed to the protein conformational disorder triggered by the stabilizing effect of lipids on proteins [43]. An increased intensity of the ^13^C NMR signal resonances from 20.3 to 31.8 ppm, which were attributed to the methyl terminal groups, aliphatic ring methylene groups and methylene groups in long alkyl chains was observed, which further supported the potential formation of the V-type amylose-lipid complex during amylolysis. 

### 3.5. Lamellar Structure

**Figure 6:**
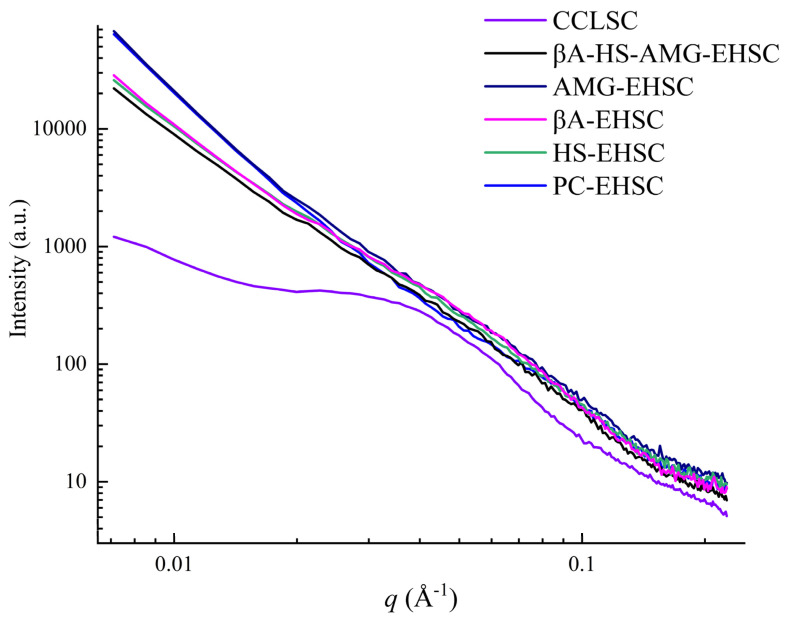
The small-angle X-ray scattering patterns of CCLSC and enzymatically hydrolyzed starch concentrate residues obtained by different enzymatic hydrolysis methods. CCLSC: lentil starch concentrates from conventionally cooked seeds; PC-EHSC: enzymatically hydrolyzed starch concentrates obtained by the treatment with pancreatin; HS-EHSC: enzymatically hydrolyzed starch concentrates obtained by the treatment with heat-stable α-amylase; βA-EHSC: enzymatically hydrolyzed starch concentrates obtained by the treatment with β-amylase; AMG-EHSC: enzymatically hydrolyzed starch concentrates obtained by the treatment with amyloglucosidase; βA-HS-AMG-EHSC: enzymatically hydrolyzed starch concentrates obtained by the treatment with β-amylase, heat-stable α-amylase and amyloglucosidase.

As opposed to XRD, which generally determines the crystallographic dimensions of native and processed starch granules associated with interhelix order, the small-angle X-ray scattering technique has been commonly used in measuring the orders along the chains with dimensions approaching 10 μm [5]. A broad scattering peak appeared over a wide *q* range centered at ca. 0.03–0.04 Å^−1^ corresponding to a ~14–22 nm semi-crystalline structure based on the Bragg’s law *D* = 2*π*/*q* was observed for CCLSC, suggesting a non-periodic structure with ordered domains randomly arranged in the amorphous matrix. The disappearance of the typical lamellar peak at 0.06–0.07 Å^−1^, resulting from both the thermal processing and enzymatic hydrolysis treatments, indicated the destruction of the alternating lamellar semicrystalline architecture of the native granular starch. From Figure 6, it can be observed that, among the EHSC samples, PC-EHSC and βA-EHSC showed an increased peak intensity at *I*_max_, viz., the intensity of the scattering maximum, whereas CCLSC exhibited an overall lower peak intensity within the studied *q* range of scattering. The relatively higher *I*_max_ was generally ascribed to the larger electron density contrast between the amorphous and crystalline regions inside the starch granules, rather than the absolute density itself. The electron density difference between the amorphous and crystalline lamellae became prominent for the EHSC samples, possibly because that the electron density of the amorphous region decreased upon amylolysis. Furthermore, as presented in Table 1, the variations in the *d_c_* value, which reflect the average thickness of the crystalline lamellae within the starch concentrate samples, resembled the trend observed for the values that represent the ordered structure of EHSC, including the C1, DD and DO values. 

Fractal analysis was carried out to provide an insight into the degree of surface smoothness and mass compactness of the EHSC. The power law exponent *α* values ranged from approximately −0.38 to −2.10 for the studied samples. Specifically, the *α* values of PC-EHSC and AMG-EHSC were less than −3, indicating that these two EHSC samples were of a surface fractal structure, in which case the surface fractal dimension *D*_s_ can be calculated as *D*_s_ = 6 + *α*, and is regarded as an indicator of the degree of smoothness of the starch concentrate granules. When the *D*_s_ value was close to 2, the surface of the scattering objects tended to be very smooth [44]. The *α* values of CCLSC, βA-EHSC, HS-EHSC and βA-HS-AMG-EHSC were in the range between −3 and −1, suggesting that these samples were characterized as a mass fractal structure, in which case the fractal dimension (*D*_m_) can be calculated as: *D*_m_ = −*α*. The *D*_m_ value was 2.10 without enzymatic hydrolysis, indicating thar the packing efficiency was relatively low for CCLSC. On the other hand, the comparatively high value of *D*_m_ (2.58) for βA-EHSC suggested a relatively compact internal structure. 

## 4. Conclusions

The present study compared the multi-scale structural characteristics of the enzymatically hydrolyzed starch concentrates obtained by the action of different types of enzymes. In comparison to CCLSC, the markedly decreased *M*_w_ and DP_n_ values upon amylolysis signified that the different enzymatic treatments could produce the starch glucan molecules with much shorter and more homogeneous molecular chains. The morphological features distinguished among different samples. The XRD analysis revealed that all EHSC samples consisted of a C_A_ + V crystalline polymorph. In particular, the V-type characteristic diffraction peaks were more obvious for samples including PC-EHSC and βA-EHSC, which was in line with their lowest DP_n_ values, suggesting that their glucan polymer chains with a relatively homogenous molecular weight distribution might be more effective in participating in the formation of the V-type inclusion complex with free fatty acids in pre-cooked lentil starch concentrates. Among the EHSC samples, PC-EHSC and βA-EHSC also showed an increased peak intensity at *I*_max_ on the SAXS profile, whereas CCLSC exhibited an overall lower peak intensity within the studied *q* range of scattering. The highest XRD crystallinity and the lowest DP_n_ value obtained for PC-EHSC indicated that the starch concentrate polymers modified by pancreatin could produce glucan chains with a comparatively homogenous *M*_w_ distribution and are readily recrystallized by hydrogen bonding through chain aggregation. Comparatively, the lowest relative crystallinity for HS-EHSC obtained from XRD suggested that thermostable α-amylolysis was unfavorable for the formation of the molecular structure with a higher degree of order in the starch concentrate samples. Both the FTIR and solid-state ^13^C CP/MAS NMR spectral features indicated the possible formation of a binary and ternary complex between amylose, protein and lipids. This study was expected to provide useful information for a deep understanding of the impact of different amylolysis actions on the structural organization of starch concentrate hydrolysates and their relevant physiological properties. 

## Figures and Tables

**Table 1:** Multi-scale structural parameters and molecular weight distribution of CCLSC and enzymatically hydrolyzed starch concentrate samples prepared by different enzymatic treatments.

Samples	Crystallinity by XRD (C1, %)	Crystallinity by ^13^CNMR (C2, %)	Double Helix Content by ^13^C NMR (%)	PPA by ^13^C NMR (%)	DO Value by FTIR	DD Value by FTIR	*d_c_* by SAXS	α by SAXS	*D_m_*	*D_s_*	Average *M_w_* by SEC-MALLS (g/mol)	DP_n_ by SEC-MALLS
CCLSC	1.06 ± 0.0169 ^d^	39.33 ± 0.8027 ^d^	31.62 ± 0.6207 ^a^	7.62 ± 0.0659 ^a^	1.0152 ± 0.0002 ^b^	0.9825 ± 0.0006 ^e^	4.68	−2.10	2.10	ND	5.142 × 10^7^	2.538
PC-EHSC	4.45 ± 0.3818 ^a^	77.92 ± 0.6977 ^a^	25.04 ± 1.0063 ^b^	8.19 ± 0.5233 ^a^	1.0196 ± 0.0005 ^a^	1.0452 ± 0.0012 ^e^	7.73	−3.18	ND	2.82	3.309 × 10^4^	1.037
AMG-EHSC	1.30 ± 0.0707 ^b^	23.26 ± 0.5650 ^e^	11.89 ± 1.2243 ^d^	6.95 ± 0.1199 ^b^	1.0167 ± 0.0002 ^b^	1.0440 ± 0.0000 ^b^	6.28	−3.16	ND	2.84	2.395 × 10^6^	1.132
βA-EHSC	0.81 ± 0.0707 ^c^	42.54 ± 0.6264 ^c^	14.63 ± 0. 5039 ^c^	4.60 ± 0.2407 ^c^	1.0147 ± 0.0011 ^d^	0.9745 ± 0.0002 ^d^	4.67	−2.58	2.58	ND	5.690 × 10^4^	1.038
HS-EHSC	0.61 ± 0.0354 ^c^	19.34 ± 0.5874 ^f^	9.87 ± 0.0056 ^d^	3.53 ± 0.0870 ^d^	1.0112 ± 0.0003 ^d^	0.9740 ± 0.0002 ^d^	4.38	−2.46	2.46	ND	1.711 × 10^3^	1.262
βA-HS-AMG-EHSC	1.30 ± 0.0778 ^b^	47.30 ± 0.5405 ^b^	15.98 ± 0.8533 ^c^	2.19 ± 0.2328 ^e^	1.0161 ± 0.0003 ^c^	0.9877 ± 0.0000 ^c^	4.87	−2.45	2.45	ND	3.957 × 10^4^	1.101

CCLSC: lentil starch concentrates from conventional cooked seeds; PC-EHSC: enzymatically hydrolyzed starch concentrates obtained by the treatment with pancreatin; AMG-EHSC: enzymatically hydrolyzed starch concentrates obtained by the treatment with amyloglucosidase; βA-EHSC: enzymatically hydrolyzed starch concentrates obtained by the treatment with β-amylase; HS-EHSC: enzymatically hydrolyzed starch concentrates obtained by the treatment with heat-stable α-amylase; βA-HS-AMG-EHSC: enzymatically hydrolyzed starch concentrates obtained by the treatment with β-amylase, heat-stable α-amylase and amyloglucosidase; PPA: proportion of amorphous phase; DO: degree of order; DD: degree of double helix; *d_c_*: thickness of crystalline layer; *α*: power law exponent; *D_m_*: mass fractal dimension; *D_s_*: surface fractal dimension; DP_n_: polydispersity index (*M*_w_/*M*_n_); ND: not determined. For a given parameter, mean values bearing different lower-case letters within the same column are significantly different (*p* < 0.05).

## Data Availability

Data is contained within the article.
